# Physicochemical Attributes of Tomatoes after Different Forms of Harvesting and Transportation for Industrial Processing

**DOI:** 10.1371/journal.pone.0319668

**Published:** 2025-04-02

**Authors:** Tulio de Almeida Machado, Haroldo Carlos Fernandes, Claudinei Martins Guimarães, Clarice Aparecida Megguer, Woska Pires da Costa, Fábio Lúcio Santos, Nerilson Terra Santos

**Affiliations:** 1 Instituto Federal de Educação Goiano - Campus Morrinhos, Morrinhos, Goiás, Brazil; 2 Universidade Federal de Viçosa, Viçosa, Minas Gerais, Brazil; 3 Universidade Federal de Lavras, Lavras, Minas Gerais, Brazil.; Universidad Autónoma Agraria Antonio Narro, MEXICO

## Abstract

**Background:**

The mechanized harvesting and transportation of tomatoes can significantly impact their physicochemical characteristics, affecting quality and industrial processing efficiency. This study aimed to evaluate the effects of harvesting methods and transportation conditions on the firmness, titratable acidity (TA), total soluble solids (TSS – °Brix), pH, and percentage of loss of fresh mass (LFM) of tomatoes intended for industrial use.

**Methods:**

The mechanized harvesting and transportation of tomatoes can significantly impact their physicochemical characteristics, affecting quality and industrial processing efficiency. This study aimed to evaluate the effects of harvesting methods and transportation conditions on the firmness, TA, TSS (°Brix), pH, and percentage of LFM in tomatoes intended for industrial use.

**Results:**

Mechanized harvesting reduced tomato firmness, TA, pH, and increased mass loss. Manual harvesting resulted in 29.7% greater firmness than mechanical harvesting. The vibration effects varied depending on the floor and direction within the container, but container type did not significantly influence tomato quality. The position of the fruit in the transport medium affected firmness, with tomatoes at the rear exhibiting greater firmness and lower quality loss. Depth was negatively correlated with firmness, LFM, TA, and pH. The LFM increased with longer unloading times.

**Conclusion:**

The findings highlight the need for improved handling and logistics strategies in the tomato production chain to reduce quality deterioration during harvesting and transportation. Effective interventions can minimize economic losses and increase industrial processing efficiency. Additionally, the results of this study suggest that laboratory models that use equipment such as shakers can replicate these effects for other bulk-transported crops, including fresh fruits and tubers.

## 1. Introduction

Tomato (*Solanum lycopersicon* L.), a member of the Solanaceae family, is a vegetable that is highly productive and highly consumed worldwide [1,2]. Worldwide tomato production totaled 161.7 million tons, with the USA contributing 13.2 million tons, but China and India occupied first and second places, respectively, in the production of this crop [3]. Several other countries on all continents produce tomatoes, such as Italy [4,5], Africa [6], Nepal [7], India [8], Korea [9], Australia [10], and Iraq [11]. It is a popular vegetable grown all over the world and is thought to have originated in the Andean area, which includes portions of Colombia, Ecuador, Peru, Bolivia, and Chile [12]. Visionary research is already underway, including the planting of tomatoes in consortium with other crops to enable the survival and long-term sustainability of colonies on Mars in the future [13]. This vegetable is especially common in Brazil [14], which presented, in the year 2022, a tomato production of 3,809,986 t in 54,502 hectares of harvested area, with an average yield of 69,905 kg per hectare, being the State of Goiás the largest tomato producer in Brazil [15].

Tomatoes are important components of many human diets and value fruit quality. However, tomatoes are highly perishable and have significant post-harvest losses [16], which need to be reduced in the crop production process, whether for fresh consumption or industrial processing. Tomato production and consumption are increasing worldwide, with production and consumption increasing with population growth [17].

Tomato production faces multiple challenges, including high input costs, pest and disease management, post-harvest losses [18], agronomic limitations, and mechanical damage, reinforcing the need for alternative control methods [19]. Among these challenges, transportation from the field to the industry is critical, as inadequate management can lead to significant economic losses. Among these challenges, transportation from the field to the industry is particularly critical, as inadequate handling can result in significant economic losses. These fruits are highly susceptible to compression and impact damage during transportation [20]. Vibration trucks generated during road transport play a key role in fruit deterioration [21], contributing to external and internal damage along the supply chain [22,23]. Consequently, the production chain requires a type of fruit that is more resistant to transport and that is cultivated in low-growing systems with simplified agricultural practices and reduced production costs.

The transportation process considerably affects fresh tomato quality. High vibration levels cause severe damage, especially as the transit time increases for certain fruit types [24,25]. Several studies have investigated the effects of vibration on fresh produce during transport [24–27], including tomatoes [28]. These studies indicate that internal damage from vibration accelerates degradation [29], affecting key attributes of tomato quality [23]. Moreover, transportation often occurs in inadequate vehicles, leading to cracked, crushed, or disintegrated fruits upon arrival at processing facilities. In addition to transport-related vibrations, mechanical injuries occur at multiple post-harvest handling stages, including mechanized harvesting, packaging, and transport [30]. In this context, fresh tomatoes are highly susceptible to improper handling, which compromises key quality parameters desired by the industry [23].

Even when mechanical damage is not visually detectable, it can stimulate ethylene production, alter coloration, and change the profile of volatile compounds, negatively impacting the sensory quality of the fruit [31]. In addition, this damage resulting from compression, impact, and injury leads to mass loss, reduced firmness, and changes in enzymatic activity, compromising tomato quality and shelf-life [32]. Damage to the fruit epidermis can weaken its natural barriers, increasing susceptibility to water loss [33]. These effects are associated with disruptions in cell wall integrity, increased water loss, and increased respiration rates [24,25]. Cell wall rupture is directly linked to turgor loss, whereas enzymatic activity within cells contributes to a progressive decline in product integrity [29]. Although a decrease in fruit firmness is a natural consequence of ripening, it is further accelerated by transport conditions that compromise cell membranes and walls [34]. The extent of damage is compounded when fruits are transported over long distances, especially on unpaved roads or poorly maintained highways, which tends to cause greater cargo vibration inside the transport containers [35–37]. Under these conditions, cargo vibration inside transport containers intensifies, leading to greater physical deterioration of the fruits [37,38].

Fresh tomatoes must have high resistance to bulk transport, uniform red coloration, and optimal levels of total soluble solids (TSS) and citric acid, among other quality attributes [39]. However, these parameters often decline during harvest and transport [40]. As climacteric fruits, tomatoes undergo significant physiological and biochemical changes post-harvest, reducing their quality [8,14,41]. Mechanical damage during harvesting and transport accelerates the maturation process [40], affecting key attributes such as titratable acidity (TA), TSS (°Brix), pH, and fresh mass loss percentage (LFM) [40]. For industrial processing, the TSS (°Brix) and pH are critical quality parameters influencing the flavor and shelf-life of the final product [20,42]. Titratable acidity also affects texture and taste [42,43], whereas color and viscosity are closely related to the sensory acceptance of processed products such as ketchup and sauces [44]. The industry closely monitors these parameters, as they directly impact product quality.

On a microscopic scale, mechanical damage to tomatoes results from cell wall failure, manifesting as macroscopic defects [33]. However, detecting and preventing microscopic injuries—such as internal tissue damage, cellular breakdown, and microcracks caused by repetitive compression and impact—remains a challenge for the food industry. Extended transportation distances and high storage temperatures exacerbate these changes, leading to increased weight loss, reduced firmness, and color alterations [35]. Variations in transportation conditions, post-harvest treatments, and storage environments significantly influence fruit quality and overall losses [45].

Post-harvest losses in tomatoes can reach up to 42% globally, underscoring the need for improved management strategies across the supply chain [46]. Studies that accurately assess post-harvest loss rates and their underlying causes are crucial for reducing waste, increasing profitability, and enhancing competitiveness for producers [47,48]. Given these considerations, the present study aimed to evaluate the impact of mechanized harvesting and transportation on the physicochemical characteristics of tomato fruits intended for industrial processing.

## 2. Materials and methods

### Cultivation area location and tomato hybrid variability

The present research was conducted at “Bom Jardim” Farm (17° 63’ 39” S longitude, 49° 07’ 36” W latitude and 773 m altitude) in the municipality of Morrinhos, GO, Brazil. The experimental area consisted of 55 ha of tomatoes for industry, cultivated under a center-pivot irrigation system, where sweet corn had previously been cultivated. The site has slightly undulating relief (10%) and predominant soil of the Dark Red Latosol type [49].

In the study area, the tomato hybrid BA5630 from BHN was transplanted and grown in a no-tillage system, and cultural practices were implemented according to recommendations for commercial cultivation.

### Harvesting and manual and mechanical transportation damage analysis

A descriptive and comparative analysis of the damage caused to tomatoes in different processes was carried out: manual harvesting, mechanized harvesting, and transportation after mechanized harvesting. This analysis aims to demonstrate the level of damage associated with each of these processes. Before mechanical harvesting, a fruit sample was collected manually for future comparisons. This treatment was considered the control (no mechanical intervention) for comparison. For fruits that underwent mechanical harvesting, sampling was carried out immediately after harvesting at the exit of the unloading arm. They did not go through the transportation situation in the truck. In these two stages, eight repetitions of each treatment were collected, with ten fruits per repetition, for later comparison with the transported fruits.

Mechanized harvesting was performed 127 days after transplanting (DAT), via a self-propelled harvester (GUARESI, model G-89/93 MS 40”, 128.7 kW FIAT-Iveco engine) with a floating collection platform and an electronic sorter of green fruits and lumps. The average soil water content at harvest was 18% [50]. The evaluated harvester’s threshing system adopted the manufacturer’s suggested configuration (12 rpm rotation and 2.5 Hz vibration frequency). During the operation, the sensor for separating clods and green fruits was turned off, and the selection of green fruits and impurities was carried out manually, directly on the harvester conveyor belt.

The mechanically harvested fruits that ended up on the truck were evaluated for damage caused by mechanized transport (the most common process for industrial tomatoes). For this analysis, a general average was used for each physicochemical attribute evaluated, regardless of the container, depth, position, and time of sample removal. This part of the experiment was carried out in a completely randomized design with four replications.

All the fruits harvested (manual, mechanized, and transported) were taken to the laboratory for evaluation of their physicochemical attribute firmness, TA, TSS (°Brix), pH, and LFM. Firmness was estimated via the flattening method [51], with involves evaluating five fruits per repetition and two measurements per fruit. The methodology determined the TA [52]. The TSS (°Brix) was estimated using a portable refractometer (Instrutemp brand, scale 0–32 °Brix). The pH was measured with a pH meter (Lucadema, model Luca 210P). To evaluate LFM, the fruits were weighed at 0, 24, 48, 72, 96, and 120 h after harvest. The LFM (% weight loss) was estimated by relating the initial fruit weight before treatment to that after the last weighing.

### Tomato transportation description

A comparative analysis of the behavior of two containers (truck bed and trailer attached to the truck) was carried out on two types of pavements (dirt and asphalt roads). This analysis aimed to evaluate how containers behave on different floors in terms of the vibrations of containers and the physicochemical damage caused during transport. For transport, a truck (Volkswagen, model 31330, 242.7 kW Cummins ISL engine, 6 × 4 traction) was used, with a truck body used to transport 40 m³ roll on/off buckets and a trailer (Imavi brand), with wheel double axles, a chassis and a shock absorber.

**Fig 1:**
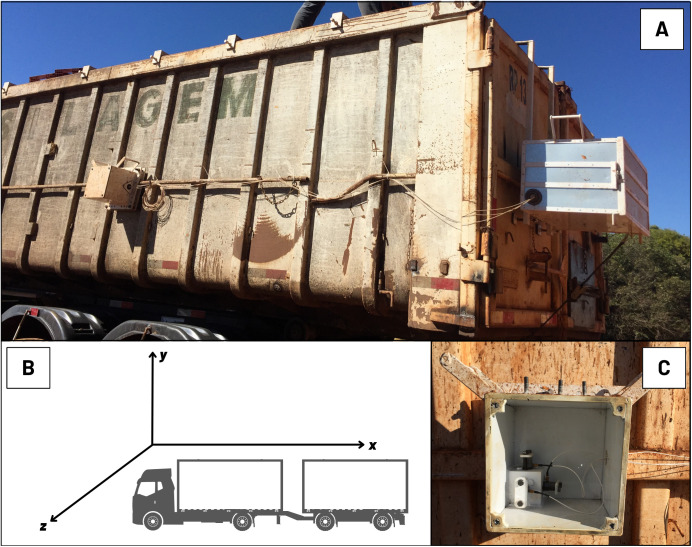
(A) Accelerometers and data acquisition system mounted in a transport container, and (B and C) arrangement of the accelerometers’ xyz axes concerning the truck’s direction. Morrinhos, GO, Brazil.

For fruit transport by the truck/trailer set, the levels of general average acceleration (GAA) (m s^–2^), which characterize the vibration treatments (asphalt pavement and dirt pavement), were evaluated through three high-sensitivity axial accelerometers (peak measurement range up to ± 490 m s^–2^). The accelerometers were arranged orthogonally, following the *xyz* axes orientations, at a point in the trailer and truck buckets to estimate the vibration level (Fig 1). For the evaluation of the transport container, which was composed of the trucks’ bucket, the accelerometers were positioned just above the rear axle, which was assumed to be the place with the highest vibration level because the impacts suffered during the displacement on the different surfaces. The accelerometers were positioned between the front and rear axles of the compost container on the trailer.

The acceleration signals were collected in the three orthogonal *xyz* axes (Fig 1) through a data acquisition system from National Instruments (model NI cDAQ-9174), with four channels connected to LabView^®^ software version 5.0 at regular intervals. During transport, the measurements were separated according to the type of pavement (dirt or asphalt road). The average travel speed of the truck was 22 km h^–1^ in journeys of 5 minutes to 10 minutes for dirt pavement and 63 km h^–1^ in journeys of 30 minutes to 35 minutes for asphalt pavement, which was classified as regular [53].

**Fig 2:**
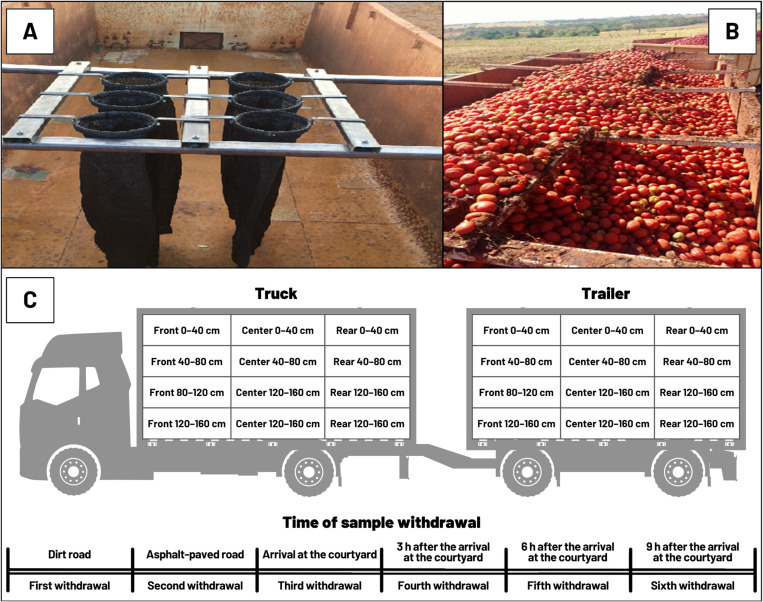
A sample of bulk tomato cargo was installed in the truck bucket before mechanized harvesting, and an experimental sample collection scheme was used during transport. Morrinhos, GO, Brazil.

In sections paved with dirt and asphalt, the root mean square (RMS) of the three axes was calculated following [54]. The RMS (Eq 1) was obtained from the mean square root of the instantaneous acceleration values, which occurred during a given measurement period.


$${\text{RMS}}_{j}={\left(\frac{\sum a_{i}^{2}}{N}\right)}^{0.5}$$ (1)

where: *a*_*i*_ is the value observed on the axis (*x*, *y*, or *z*) at time *i* (*i* =  1, 2,..., *N*); *N* is the number of observations on the axis (*x*, *y*, or *z*); and *j* represents the specific axle (*x*, *y*, or *z*). Therefore, RMS*x* is the root mean square value of the *x*-axis, RMS*y* is the root mean square value of the *y*-axis, and RMS*z* is the *z*-axis root mean square value. Three replicates were collected from each axle (*xyz*) for each transport container. A fruit sample collector for bulk-transported cargo developed by [55] was installed in buckets to remove fruits from the transport system (Fig 2).

All the fruits harvested (manual, mechanized, and transported) were subsequently taken to the laboratory to evaluate the physicochemical attributes of firmness, TA, TSS (°Brix), pH, and LFM. Firmness was estimated through the flattening method [51], which involves evaluating five fruits per repetition and two measurements per fruit. The methodology determined TA [52]. The TSS (°Brix) was estimated via a portable refractometer (Instrutemp brand, scale 0–32 °Brix). The pH was measured with a pH meter (Lucadema, model Luca 210P). For LFM (% weight loss), the fruits were weighed at 0, 24, 48, 72, 96 and 120 h after harvest, and the weight was estimated on basis of the initial fruit weight before treatment and after the last weighing.

This second part of the experiment (transport) was conducted in a completely randomized design in sub-sub-subdivided plots, with 144 treatments and four replications for each experimental unit (Fig 2). The treatments included two types of transport containers, three horizontal positions of the sample collector inside each transport container, four depths in each position, and six different moments of sample withdrawal [56].

Sub-sub-subdivisions used: (A) Level of plots: Transport container (truck or trailer); (B) Level of sub-plots: Position of the collectors (front, central, and rear); (C) Level of sub-sub-plots: Depth levels (0–40 cm, 40–80 cm, 80–120 cm, 120–160 cm); and (D) Level of sub-sub-sub-plots: Time of sample withdrawal (dirt road, asphalt pavement, arrival at the yard, 3 hours after arrival at the yard, 6 hours after arrival at the yard; 9 hours after arrival at the yard).

### Statistical analysis

The data were subjected to analysis of variance (ANOVA), and the means were compared by Tukey’s test, at 5% probability (*p* ≤ 0.05), using the SISVAR software [57]. Together with the compared means, the following were added: the standard error (*SE*) when there were several different samples and the standard deviation (*SD*) for the number of equal samples.

## 3. Results and discussion

### Mean squared acceleration (RMS) in each axis analyzed

For the dirt paving, considering the z axle, the tomato fruits transported in bulk by the trucks bucket experienced the highest vibration levels, which were 59.9% higher than the load transported by the trailer. For asphalt paving, the fruits transported in the trailer bucket presented the highest acceleration levels (Table 1).

**Table 1:** RMS (m s^–2^) of acceleration for different axes, tomato containers, and paving types.

Container	Axle *z*		Axle *y*		Axle *x*	
	Dirt	Asphalt	Dirt	Asphalt	Dirt	Asphalt
**Truck (C1)**	1.225 a	0.915 a	0.302 a	0.436 b	0.360 a	0.524 a
**Trailer (C2)**	0.766 b	1.021 a	0.345 a	0.670 a	0.406 a	0.787 a

**Note:** The study was conducted with hybrid BA5630 tomatoes (BHN) in Morrinhos, GO, Brazil. C1: container 1 (Truck); C2: container 2 (Trailer); means followed by the same letter in the same column do not differ statistically by Tukey’s test at 5% probability (*p* ≤ 0.05).

The displacement speed of the transport set (truck/trailer) was lower on the dirt stretch, where the trend was that the containers with more stable bases (two supports) presented the lowest levels of vibration, a result that can be observed on the *y*-axis (Table 1). However, for the same axis (*y*), on the asphalt road with the highest displacement speed, the tomato transport container contained in the trailer was affected by the resulting average acceleration values since the trailer was supported on two axles, being joined to the truck using a pin contained in its header, causing the gap of this union to affect the vibration values in situations of acceleration and braking of the conveyor assembly at higher speed.

With longer travel times and transport speeds on asphalt, these results (Table 1) corroborate those of [58]; who reported that the lack of protection on trucks to avoid the transmission of vibrations and poorly paved roads are some of the factors that directly influence the qualitative losses of agricultural products during transport.

### Damage during manual and mechanical harvesting and transportation

There was a significant difference between treatments for all physicochemical attributes studied for tomato, except for TSS, indicating that the insertion of mechanized steps during the process contributed to changes in the characteristics analyzed (Table 2).

**Table 2:** Analysis of variance (ANOVA) and mean values with *SE* for firmness, TA (% of citric acid), TSS (°Brix), pH, and LFM (% weight loss) of tomatoes subjected to different treatments.

*FV*	*DF*	Firmness		TA		TSS (°Brix)		pH		LFM	
		*MS*	*F*_calc_	*MS*	*F*_calc_	*MS*	*F*_calc_	*MS*	*F*_calc_	*MS*	*F*_calc_
**Trat**	2	1.137	5.58^*^	0.015	7.30^*^	0.049	0.26^ns^	0.160	17.04^*^	147.639	23.52^*^
**Res**	589	0.204		0.002		0.189		0.009		6.278	
**Total**	591	0.207		0.002		0.189		0.010		6.757	
**Trat**		**Firmness (N cm**^**–2**^)		**TA (%)**		**TSS (°Brix)**		**pH**		**LFM (%)**	
**Trat1**		2.14 ± 0.26 a		0.34 ± 0.00 b		3.83 ± 0.14 a		4.79 ± 0.02 a		4.40 ± 0.50 b	
**Trat2**		1.65 ± 0.21 ab		0.37 ± 0.01 ab		3.96 ± 0.09 a		4.61 ± 0.01 b		9.13 ± 0.71 a	
**Trat3**		1.60 ± 0.01 b		0.40 ± 0.00 a		3.93 ± 0.01 a		4.59 ± 0.00 b		10.40 ± 0.10 a	

**Note:** Treatments included hand harvesting, mechanical harvesting, and post-harvest transportation. The study was conducted with hybrid BA5630 tomatoes (BHN) in Morrinhos, GO, Brazil. *FV*: factor of variation; *DF*: degrees of freedom; *MS*: mean square; *F*_calc_: *F* calculated; Trat: treatments; Res: residue; Trat1: hand harvest; Trat2: mechanized harvest; Trat3: transported;

* : significant at 5% probability (*p* ≤ 0.05) according to the *F* test, ^ns^: not significant at 5% probability by *F* test; means followed by the same letter, in the column, do not differ statistically from each other, according to Tukey’s test at 5% probability.

In terms of the firmness of the tomato fruit, firmness of the Trat1 group was 29.7% greater than that of the Trat2 group, and the firmness of the Trat3 group was 33.7% greater than that of the Trat3 group. On the other hand, the mean firmness of the mechanically harvested fruits was intermediate between those of the Trat1 and Trat3 (Table 2). This result indicates that fruit firmness tends to decrease with mechanical intervention. Inverse results for the treatments were observed for TAs. The three treatments detected in no significant differences in SST (°Brix).

Manual harvesting reduced LFM by 51.8%, increased the pH by 3.9%, and caused less mechanical damage to the fruit than the other treatments did (Table 2). Mechanical intervention promotes injuries, even if internal, resulting in reduced fruit firmness [30], which is evidenced by the results for the percentage of LFM on the evaluated samples. With increased damage, the fruits tend to increase ethylene production and the respiratory rate [40].

When bound to its receptor site, ethylene is a gaseous phytohormone that triggers a series of reactions and, in cases of physical damage, stimulates the production of enzymes that degrade cell wall compounds, such as cellulases and pectinases, thus reducing pulp firmness [40]. When the fruit suffers physical damage, it activates mechanisms to seal the wound, increasing respiratory activity, and, in return, there is consumption of a reserve substance such as sucrose, also leading to an increase in the production of citric acid in the Krebs cycle, which explains the increase in TA in fruits that suffer more significant mechanical injury [59].

The results in Table 2 corroborate those of [60], who, when evaluating two varieties of peach (*Prunus persica* L. Batsch) concerning injuries, concluded that fresh fruits generally suffer mechanical damage during harvesting, transport, and storage and that physical injuries are important in abiotic stress, which can significantly compromise fruit quality and shelf life, as well as trigger a series of responses from physiological and pathological factors.

The most significant losses in collected fruits were observed after the resting phase, at the end of processing, and at the marketing center, as identified in a study on the LFM of ‘Tahiti’ acid limes [61]. These losses are attributed to the increased handling of the fruits, which subjected them to more extensive physical damage than those not passing through the processing line.

Although manual harvesting causes less physicochemical damage than mechanical harvesting does, an economic viability and process efficiency analysis must be carried out in each case since mechanization is the most common way of harvesting and transporting tomatoes for industrial processing.

### Physicochemical attributes in transport

There was statistical significance for the factors position (firmness), depth (TA and °Brix), and time of sample withdrawal (TA, pH, and LFM), in addition to the interactions depth versus container (LFM), depth versus position (pH) and time of withdrawal versus container (firmness and pH) (Table 3).

**Table 3:** Analysis of variance (ANOVA) for container (C), position (P), depth (D), and removal time (T) factors and their interactions in terms of average attributes of firmness, AT, TSS (°Brix), pH, and LFM for tomato.

*FV*	*DF*	Firmness (N cm^–2^)		TA (%)		TSS (°Brix)		pH		LFM (%)	
		*MS*	*F*_calc_	*MS*	*F*_calc_	*MS*	*F*_calc_	*MS*	*F*_calc_	*MS*	*F*_calc_
**C**	1	2.996	0.58 ^ns^	0.001	0.01 ^ns^	12.076	1.85 ^ns^	0.004	0.01 ^ns^	359.567	1.91 ^ns^
**Res (C)**	6	5.135		0.057		6.525		0.087		188.132	
**P**	2	0.574	6.03^*^	0.003	1.64 ^ns^	0.209	1.34 ^ns^	0.008	1.04 ^ns^	0.805	0.09 ^ns^
P*C	2	0.166	1.75 ^ns^	0.001	0.64 ^ns^	0.016	0.10 ^ns^	0.005	0.70 ^ns^	0.275	0.03 ^ns^
**Res (P)**	12	0.095		0.002		0.156		0.007		9.214	
**D**	3	0.411	1.83 ^ns^	0.064	31.26^*^	0.581	2.82^*^	0.059	6.91^*^	65.138	17.82^*^
D*C	3	0.028	0.12 ^ns^	0.002	1.20 ^ns^	0.164	0.80 ^ns^	0.008	0.87 ^ns^	18.420	5.14^*^
D*P	6	0.198	0.88 ^ns^	0.001	0.62 ^ns^	0.102	0.50 ^ns^	0.028	3.12^*^	3.556	0.99 ^ns^
**Res (D)**	54	0.225		0.002		0.206		0.009		3.586	
**T**	5	0.305	2.36^*^	0.002	2.40^*^	0.033	0.38 ^ns^	0.086	11.86^*^	12.926	4.21^*^
T*C	5	0.397	2.71^*^	0.002	1.57 ^ns^	0.070	0.81 ^ns^	0.016	2.24^*^	4.520	1.47 ^ns^
T*P	10	0.100	0.68 ^ns^	0.000	0.46 ^ns^	0.079	0.91 ^ns^	0.011	1.46 ^ns^	4.918	1.60 ^ns^
T*D	15	0.067	0.46 ^ns^	0.002	1.65 ^ns^	0.076	0.87 ^ns^	0.004	0.58 ^ns^	4.166	1.36 ^ns^
**Res (T)**	390	0.147		0.001		0.087		0.007		3.074	
**Total**	575	0.198		0.002		0.191		0.010		6.357	

**Note:** The study was conducted with hybrid BA5630 tomatoes (BHN) in Morrinhos, GO, Brazil. *FV*: factor of variation; *DF*: degrees of freedom; *MS*: mean square; *F*_calc_: *F* calculated; Res: residue;

* : significant at 5% probability (*p* ≤ 0.05) according to the *F* test, ^ns^: not significant at 5% according to the *F* test.

The *F* test from the ANOVA was conclusive for significant factors. The average firmness was statistically lower for the front position (6.0% lower) about the rear, whereas the middle position presented a statistically intermediate value (1.59 ± 0.45) between the two (Table 4). As the tomato load was transported in bulk and not in boxes, the fruit distribution was not uniform inside the transport container, keeping the container ends with lower loading heights.

**Table 4:** Means and *SD*s for firmness, TA (% citric acid), TSS (°Brix), and LFM (% weight loss) for tomatoes in different positions (P), depths (D), and times of sample withdrawal (T) during transport.

Factor	Firmness (N cm^–2^)	TA (%)	TSS (°Brix)	LFM (%)
**P1**	1.56 ± 0.41 b	–	–	–
**P2**	1.59 ± 0.45 ab	–	–	–
**P3**	1.66 ± 0.45 a	–	–	–
**D1**	–	0.38 ± 0.03 c	3.90 ± 0.45 a	–
**D2**	–	0.39 ± 0.03 c	3.89 ± 0.41 a	–
**D3**	–	0.41 ± 0.04 b	3.90 ± 0.43 a	–
**D4**	–	0.43 ± 0.05 a	4.02 ± 0.44 a	–
**T1**	–	–	–	9.84 ± 2.34 b
**T2**	–	–	–	10.51 ± 2.66 ab
**T3**	–	–	–	10.15 ± 2.33 b
**T4**	–	–	–	10.35 ± 2.80 ab
**T5**	–	–	–	10.52 ± 2.24 ab
**T6**	–	–	–	10.98 ± 2.42 a

**Note:** The study was conducted with hybrid BA5630 tomatoes (BHN) in Morrinhos, GO, Brazil. P1: front; P2: medium; P3: rear; D1: 00–40 cm; D2: 40–80 cm; D3: 80–120 cm; D4: 120–160 cm; T1: dirt road; T2: asphalt road; T3: arrival at the courtyard; T4: three hours after the truck arrives at the yard; T5: six hours after the truck arrives at the yard; T6: nine hours after the truck arrived at the yard; Means followed by the same letter, in the column, for each factor, do not differ statistically from each other, according to Tukey’s test at 5% probability (*p* ≤ 0.05). The cells with missing values (filled with a hyphen) represent the non-significance of the factor (in the line) for the variable in question (in the column).

As there is an inclination when leaving the buckets on the floor of the industry yard, both the buckets that are coupled to the truck and the buckets that are coupled to the trailer, there is a need to initially incline the container for this uncoupling to be carried out. As there were fewer fruits at the back of the transport container, there is the possibility of surface runoff of tomato fruits without them falling on the patio, causing differences in firmness across the different positions (Table 4).

The percentage of citric acid, which serves as a parameter for the measurement of TA, increased in a manner directly proportional to the depth of sample withdrawal (Table 4), generating an increase of 13.2% from the top to the bottom layers. The °Brix values remained statistically identical at the different depths studied.

The tomato TA increased with increasing loading depth inside the transport container. A greater number of damaged fruits after mechanical harvesting, together with the compression of the fruit column, with increasing depth, generated an increase in the number of damaged fruits and, consequently, the respiration rate and TA at greater load depths. Evaluating the quality of ‘Fuji Suprema’ apples subjected to different types of mechanical damage, [62] reported that damage caused by cutting provided fruits with lower TAs, considering the evaluation nine days after damage application.

The percentage of citric acid in the Tas ranged from 0.39 to 0.40 at different withdrawal times for the evaluated samples (Table 4). The *SD* remained at approximately 0.04, representing slight variation between the samples. However, the moment of withdrawal did not significantly change the AT, indicating that, in this condition, the physical attributes were more affected than the chemical attributes were.

In the significant interactions (Table 5), no significant differences were observed for firmness between the different withdrawals when truck transport was used. However, there were differences in firmness when trailer transport was used, with a greater value (1.85 ± 0.48 N cm^–2^) occurring six hours after the truck arrived at the yard, which was 19.4% greater than the lower value (1.55 ± 0.44) for the moment of arrival at the courtyard (Table 5). The average acceleration values on axle *z* (Table 1) were greater in the trailer (59.9% greater), covering most of the transport time on the asphalt road than on the dirt.

**Table 5:** Means and *SD*s of firmness, pH, and LFM for significant interactions between container (C) versus time of sampling (T), position (P) versus depth of sampling (D), and (C) versus (D) for tomato, hybrid BA5630 from BHN, during transport.

Attribute	Factor	Time of sample withdrawal							
		T1	T2		T3	T4	T5		T6
**Firmness** **(N cm**^**–2**^)	C1	1.53 ± 0.32 a	1.49 ± 0.30 a		1.56 ± 0.29 a	1.53 ± 0.54 a	1.52 ± 0.34 a		1.54 ± 0.36 a
	C2	1.62 ± 0.45 bc	1.66 ± 0.50 abc		1.55 ± 0.44 c	1.80 ± 0.55 ab	1.85 ± 0.48 a		1.58 ± 0.46 bc
**pH**	C1	4.60 ± 0.07 a	4.60 ± 0.09 a		4.61 ± 0.10 a	4.58 ± 0.08 a	4.58 ± 0.07 a		4.57 ± 0.09 a
	C2	4.62 ± 0.10 a	4.64 ± 0.12 a		4.62 ± 0.09 a	4.54 ± 0.07 b	4.54 ± 0.09 b		4.56 ± 0.09 b
		**Depth**							
		**D1**		**D2**		**D3**		**D4**	
**pH**	P1	4.60 ± 0.10 ab		4.62 ± 0.09 a		4.56 ± 0.09 b		4.58 ± 0.10 ab	
	P2	4.58 ± 0.10 a		4.63 ± 0.07 a		4.59 ± 0.09 a		4.59 ± 0.09 a	
	P3	4.61 ± 0.09 a		4.60 ± 0.08 a		4.58 ± 0.09 ab		4.53 ± 0.08 b	
**LFM (%)**	C1	11.39 ± 2.26 ab		10.57 ± 2.28 b		11.00 ± 2.62 ab		11.74 ± 3.15 a	
	C2	8.92 ± 1.61 b		8.75 ± 1.34 b		10.00 ± 2.03 a		10.77 ± 2.03 a	

**Note:** The study was conducted with hybrid BA5630 tomatoes (BHN) in Morrinhos, GO, Brazil. C1: truck; C2: trailer; P1: front; P2: medium; P3: rear; T1: dirt road; T2: asphalt road; T3: arrival at the courtyard; T4: three hours after the truck arrives at the yard; T5: six hours after the truck arrives at the yard; T6: nine hours after the truck arrives at the yard; D1: 00–40 cm; D2: 40–80 cm; D3: 80–120 cm; D4: 120–160 cm; Means followed by the same lowercase letter, on the same line, do not differ statistically from each other, according to Tukey’s test at 5% probability (*p* ≤ 0.05).

pH accompanied the physical variation in fruit firmness for the different transport containers (Table 5), differing from firmness only when the samples were transported in the trailer (reduction of 2.2% from the higher value, on the asphalt-paved road, to the lower value, three hours and 6 hours after the truck arrived at the yard). A longer exposure time of the load to vibrations on the asphalt road caused injuries to the fruits, and with a waiting time of approximately nine hours until the collection of the last samples, there was a change in pH.

Generally, the average pH values were greater in the containers’ superficial layers of the tomato load for the front and rear positions (Table 5). In the central position, all the values were similar. The superficial layers of the load had higher pH values because, in this location, there were fewer injuries caused by fruit compression. LFM evidenced this behavior in the evaluated transport containers, where the highest percentages of LFM were found at greater depths.

The percentage of LFM increased with increasing sample depth in the transport containers (Table 5), especially in the trailer container, which presented 20.7% more LFM in the 120–160 cm depth layer than in the first layer at the top (00–40 cm). The fruits in the deeper layers underwent a more extended period under compression, with greater abrasion between their epidermis, generating greater fruit water loss. The *SD* was approximately ± 2.40, indicating significant variation among the collected samples.

Change in fresh matter can occur due to water loss by fruit transpiration, and fruit resistance to moisture loss can be reduced with increasing physiological maturity [63]. Tomatoes are quite susceptible to rapid water loss, as determined by LFM, because of their thin epidermis, which has little resistance to mass transfer [64]. A study carried out on cherry tomatoes reported LFM of up to 10% after 25 days of storage at 5°C and 80 to 85% RH [65], corroborating the findings of the present study (Table 5). LFM values of approximately 10% are tolerated in natural commercialization; however, in tomatoes for industry, this loss is believed to be not visually relevant but indicates that mechanical damage is decisive in the greater loss of product mass.

Evaluating mechanical injuries in ‘Tommy Atkins’ mangoes, a study revealed a greater damage area in the epidermal layer of fruits that experienced abrasion [66]. The epidermis is a natural barrier and protects against moisture loss from internal tissues to the environment. When the epidermis ruptured, the parenchyma cells of the outermost layers of the fruits became dehydrated, reflecting an increase in fresh mass loss.

### Study strengths and limitations

As with all scientific work, this study has several limitations. First, a single tomato variety was analyzed, which restricts the generalization of the results to other varieties. Second, the data were collected for a single season and location, making it impossible to assess seasonal variations or effects under different climatic conditions. Finally, the analysis was carried out on specific routes and conditions, which may limit the applicability of the results to other regions, distances, or types of routes.

On the other hand, although some variables are known empirically, our work pioneered the scientific investigation of these variables, such as the impact of vibration on bulk tomato transportation under real conditions. This investigation made it possible to generate concrete data on the resistance and damage caused to fruit during transportation. The adopted approach reflected the conditions often encountered in the agricultural sector, indicating the results practical relevance to the use of the results. The use of laboratory accelerometers to measure vibrations and impacts has confirmed and validated previously known but not yet quantified physical data.

## 4. Conclusions

Our study assessed the impact of the transportation of mechanically harvested tomatoes on the physicochemical characteristics of fruits destined for industrial processing. The effects of vibration throughout the process, considering factors such as road conditions, inadequate (nonrefrigerated) vehicles, the environment, and the waiting time for unloading, resulted in a reduction in firmness and influenced the fruit’s TA, pH, and LFM percentage. These alterations compromised the quality of the tomatoes, contributing to quality losses and negatively impacting the cost of the raw material for the industry. Although variations in vibrations were identified between the samples in the containers and those in the truckload, the different types of containers used had no significant influence on the physicochemical quality of the fruit. On the other hand, the position in the means of transportation affected the quality of the tomatoes, with less deterioration (greater firmness) observed at the back of the vehicle. Depth also influenced parameters such as firmness, LFM, AT, and pH, indicating a negative correlation between these variables. In addition, LFM increased with waiting time for unloading. Thus, on the basis of the data collected, the method applied has the potential for developing laboratory models using equipment such as shakers, making it possible to replicate the results for other crops transported in bulk, including different types of fresh fruit and tubers. We conclude that these findings reinforce the need to implement effective handling and logistics strategies throughout the tomato production chain to minimize he economic impacts of harvesting and transport on fruit quality. These measures can contribute to a reduction in losses and greater efficiency in the industrial processing of tomatoes.

### Future research directions

Future research could explore the impact of vibration on different varieties of fruit and tubers transported in bulk, investigating cultivars and variabilities best suited to logistical and environmental conditions, types of containers, and vehicle damping systems. Replicating this experiment at different seasons could generate valuable information on the effects of seasonal factors, such as rainfall and high temperatures, on transportation and product quality, providing advantageous indicators for agribusiness decision-making.

In addition, the data obtained can be used to create vibration simulator models, allowing replication and control of different impact levels. This would result in a robust and standardized database, facilitating future analyses. Another relevant direction is the use of sensors connected to real-time monitoring systems based on digital technologies to evaluate and mitigate variables related to raw material logistics. This approach can help reduce costs, minimize food waste, and increase sustainability throughout the production chain.

## Supporting information

S1 Data Dataset. (CSV)
